# Comparison of kinematic manipulability in baseball hitting at different hitting points

**DOI:** 10.1038/s41598-025-25663-4

**Published:** 2025-11-24

**Authors:** Akio Morimoto, Takahiko Sato, Akinori Nagano

**Affiliations:** 1 Graduate School of Sports and Health Science, Ritsumeikan University, Kusatsu Shiga, Japan; 2 Faculty of Rehabilitation, Biwako Professional University of Rehabilitation, Hikone Shiga, Japan; 3 Institute of Advanced Research for Sport and Health Science, Ritsumeikan University, Kusatsu Shiga, Japan; 4 College of Sports and Health Science, Ritsumeikan University, Kusatsu Shiga, Japan

**Keywords:** Baseball hitting, Kinematic manipulability, Kinematic manipulability ellipsoid, Kinematics, Anatomy, Engineering

## Abstract

In baseball hitting, batters need to manipulate their bat positions based on the pitch location. To achieve this, batters must be capable of displacing the bat across a wide range of positions. However, no study has yet quantitatively evaluated this ability. Therefore, this study aims to assess the manipulability of bats when they hit a ball at different points using kinematic manipulability indices. The three-dimensional motions of 20 collegiate baseball players hitting stationary balls were recorded using a motion-capture system. Each participant’s body was modeled as a system of 10 connected rigid bodies with 21 degrees of freedom, from which kinematic manipulability indices were calculated using measured joint angles and segment lengths. Statistical analysis revealed that, when hitting lower or more inside locations, the bat can be further displaced horizontally and across a wider range. By contrast, the vertical component of kinematic manipulability did not vary significantly across the hitting points, thus indicating that the batters’ ability to manipulate the bat vertically might be relatively uniform regardless of the hitting location. The horizontal component was greater than the vertical component, thus suggesting that the batters are more capable of adjusting their bat position horizontally than vertically.

**Supplementary Information:**

The online version contains supplementary material available at 10.1038/s41598-025-25663-4.

## Introduction

In baseball, it is desirable for batters to hit a pitched ball hard in order to score more points than the opposing team^1,2^. The batters need to appropriately displace the bat to the location at which the pitch arrives. In addition, different types of pitches (fastballs, curves, etc.) can arrive at different locations in the strike zone. Previous research on the bat displacement at different strike locations has focused on the differences in joint angles and joint torque at different strike locations^3,4^. Although some studies have examined the kinematics and kinetics of hitting, no study has investigated the extent to which batters can displace bats. Since batters with a larger bat displacement range may establish contact with diverse pitches more effectively, this range should be quantitatively assessed as it may serve as a valuable indicator of their ability to establish contact. In robotics, the concept of, “kinematic manipulability” introduced by Yoshikawa^5^ is used to evaluate the ease of controlling the end-effector. The kinematic manipulability index of the given robot poses can be assessed as a quality value indicating the capability of adjustments in the workspace. Some researchers also proposed a methodology that considers joint torque, closed-loop system, human musculoskeletal system, human central nervous system and free-floating multi-arm system^6–9^. The use of kinematic manipulability to determine the optimal postures of various systems as well as the human body has been discussed^6,10–12^. The kinematic manipulability ellipsoid is a sphere centered on the position of the end-effector that represents the range of displacement of the end effector in the infinitesimal angular displacement of the system. The direction of the longest principal axis in the kinematic manipulability ellipsoid indicates the direction in which the end-effector is largely displaced. The kinematic manipulability measure is proportional to the volume of the kinematic manipulability ellipsoid. These kinematic manipulability indices were calculated from a kinematic perspective. Previous studies have shown that the joint angles of the batter vary depending on the impact points^3,4^. Therefore, the shape and size of the kinematic manipulability ellipsoid depend on the impact points. The objective of this study was to compare the kinematic manipulability of baseball hits at different points. Impact points with high kinematic manipulability make it easier to hit a ball because batters can easily displace the sweet spot of the bat. The knowledge gained from this study can help formulate a strategy for the batter to hit a ball in the strike zone.

## Method

### Experimental setup and data collection

The participants were 20 collegiate baseball field players (18.8 ± 0.6 years old, body height: 1.76 ± 0.05 m, body mass: 72 ± 6.2 kg). There were 16 right-handed and 4 left-handed batters. The Human Ethics Committee of Ritsumeikan University approved all study procedures (ID: BKC-LSMH-2021-034) and all participants reviewed and signed an informed consent form. All experiments were performed in accordance with the Declaration of Helsinki.

**Fig. 1:**
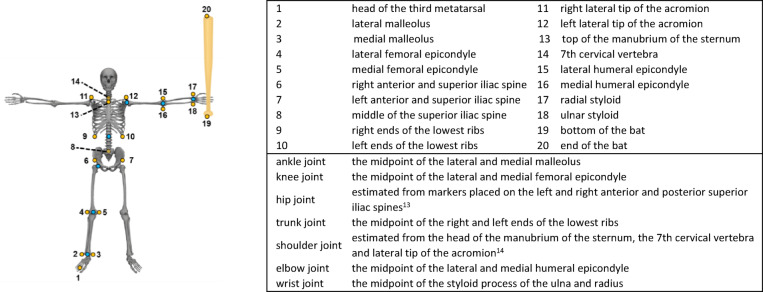
Positions of the attached reflective markers and defined joints.

**Fig. 2:**
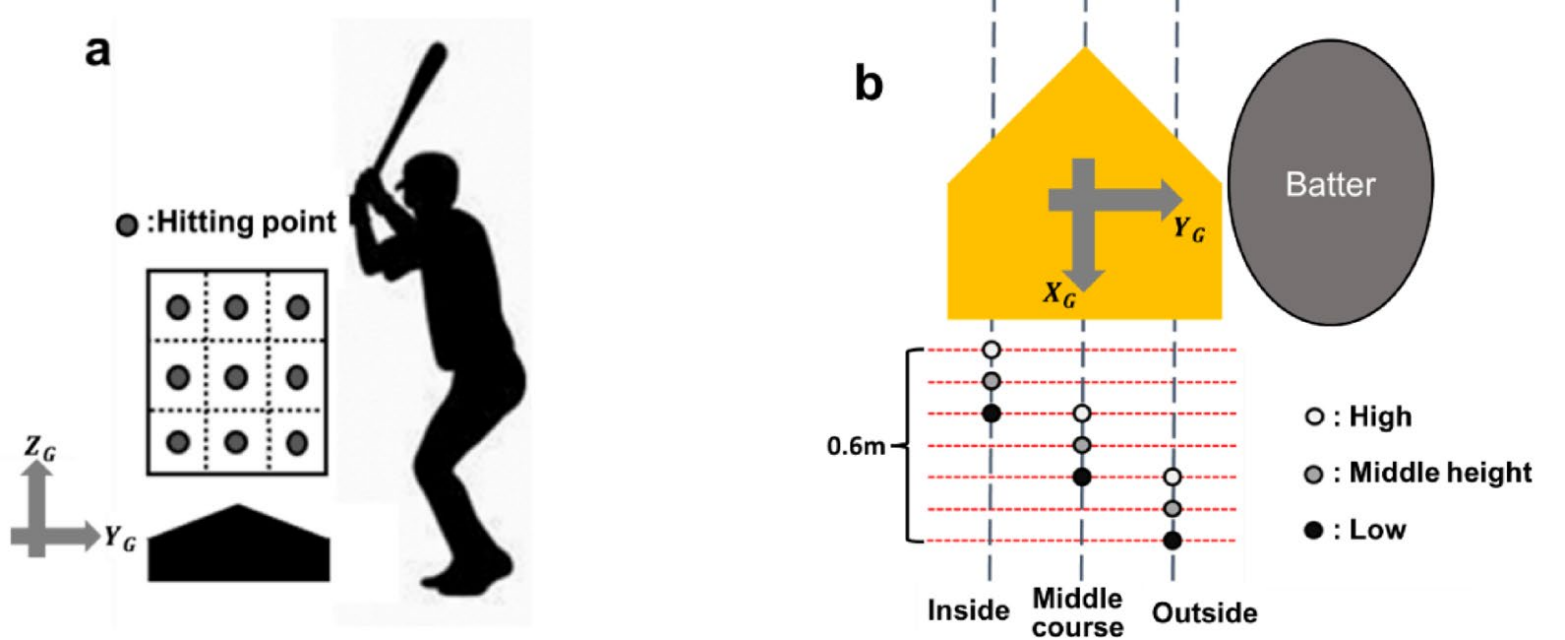
The position of hitting points.

Twenty reflective markers were attached to anatomical landmarks of each participant’s body and bat, allowing us to define seven joint locations (Fig. 1). Participants stood with their arms outstretched, and the three-dimensional positions of reflective markers were initially recorded. A ground coordinate system was defined (Fig. 2a, b), where $\:{X}_{G}$ was directed from home plate to the pitcher’s plate, $\:{Z}_{G}$ was vertically upward, and $\:{Y}_{G}$ was the cross product of $\:{Z}_{G}$ and $\:{X}_{G}$.

Yellow points are attached reflective markers. Blue points are defined joints. The attached reflective markers and defined joints are below.

In this study, tee batting was employed to precisely define the hitting point. When hitting a pitched ball, variations in ball speed and trajectory make it difficult to accurately control the hitting point location. In contrast, when hitting a stationary ball, the hitting point can be precisely controlled, which makes it possible to evaluate manipulability at each specific hitting point. In this study, the ball was placed at nine different positions, consisting of three heights (high, middle height, low) and three courses (inside, middle course, outside). Previous studies have reported that the position of the hitting point in the $\:{X}_{G}$ direction varies by approximately 0.6 m depending on the height and course^3,17^. Based on these findings, the ball was placed at different positions in $\:{X}_{G}$ depending on height and course (Fig. 2b). To confirm placed ball position, the ball was marked with two reflective markers across its diameter. The error of the midpoints of two reflective markers was within 0.03 m in $\:{X}_{G},{Y}_{G},{Z}_{G}$ for all hitting conditions. These indicate that the batter hit a distinct ball in each condition.

**a**: hitting points from pitcher’s view. **b**: hitting points from the top view. The hitting points were nine different points, consisting of 3 heights (high, middle height, low) and 3 courses (inside, middle course, outside).

In this experiment, each participant performed a total of 27 trials (3 trials per hitting point). The ball locations for hitting were presented in a randomized order, and the participants were instructed to hit each ball placed on the tee with full effort, as they would in an actual game. After each trial, the participants self-evaluated their performance on a five-point scale (1 = bad, 5 = good) and visual inspection confirmed that most of the batted balls were linear shots. As manipulability indices are based on kinematics, one should use data that directly reflect each player’s unique swing characteristics. At each hitting point, the highest self-evaluation was 4 or 5, thereby suggesting that these trials best captured each player’s individual attributes. In this study, it was assumed that the trial with the highest self-evaluation at each hitting point reflected the individual characteristics of the player, and the trial with the highest self-evaluation at each hitting point was selected for analysis.

The three-dimensional position data of the reflective markers were recorded using a 16-camera motion capture system at 1,000 Hz (Raptor-E, Motion Analysis Corporation, Santa Rosa, CA, USA). The raw data of the reflective markers were filtered using a fourth-order Butterworth low-pass filter with a cutoff frequency of 10–15 Hz, as determined in the residual analysis^18^.

## Modeling of hitting motion

In this study, the human body was modeled using a series of rigid links connected by multiple joints. This model was based on the captured kinematic data of a baseball hitting motion. The kinematic manipulability of the bat’s sweet spot in the baseball hitting motion was quantified using the kinematic manipulability indices calculated from this model. The degree of freedom (DoF) in the model were defined based on the functional capabilities of each joint in the human body. Therefore, the kinematic manipulability can be realistically evaluated using a batter. Additionally, previous studies demonstrated that a person required 100–110 ms to accelerate their hand after visual recognition^13,14^. In this study, kinematic manipulability indices were calculated based on the posture of the model 100 ms before the moment of bat-ball impact while considering the time required for a person to accelerate their body after visual recognition. This moment represents the final moment at which a batter can adjust the bat’s position based on visual feedback. Thus, high kinematic manipulability at this moment indicates that the batter possesses a greater bat-control range.

**Fig. 3:**
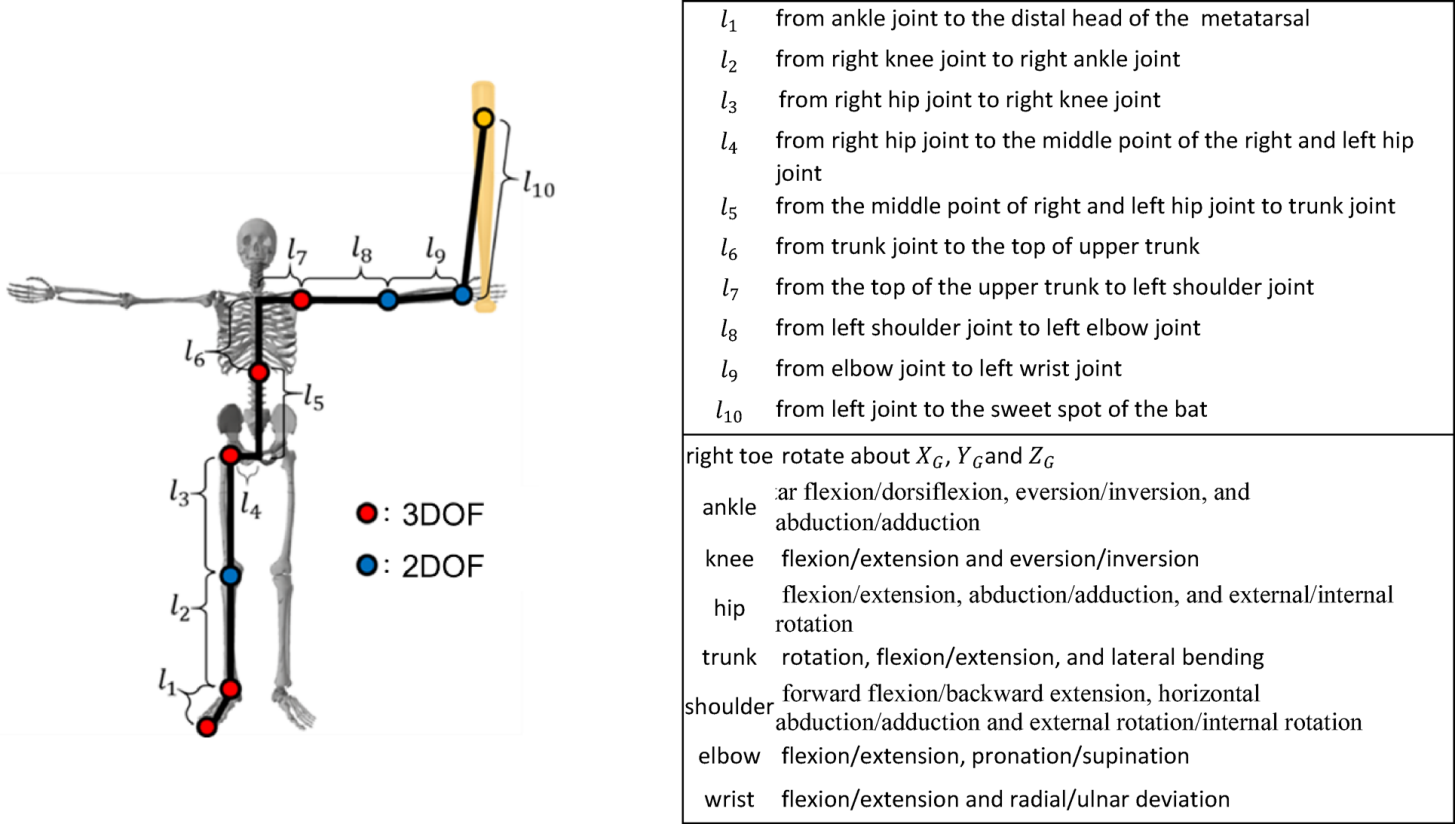
Rigid linkage model for right-handed batters.

A rigid-linkage model with 10 segments and 21 DoF was constructed (Fig. 3). Either the right or left limb was modeled for the construction of the rigid-linkage model. The leg on the catcher’s side and the arm holding the grip end of the bat were modeled. This model focused on the lead arm of the batter. The trailing arm was excluded from the rigid-linkage model to reduce complexity. Previous studies have reported that joint angles of the lead arm differ depending on the hitting location^3,4^. While we acknowledge that this assumption is a simplification, adopting this approach allows us to apply a serial-link manipulability model, which is well-established in robotics, to the batter’s arm. The DoF of each joint was determined based on the characteristics of human joints. The foot could rotate about the toe with respect to the ground. The length of each segment is denoted as $\:{l}_{1},\:{l}_{2},\:\:\cdots\:,{l}_{10}$. These lengths were calculated based on the static standing posture of the participants (Fig. 3). To perform the hitting motion, each joint angle in the hitting experiment was substituted with the joint angle of this model.

For left-handed batters, this model is the opposite for leg and arm. The length of each segment and rotation axis of each link are below.

**Fig. 4:**
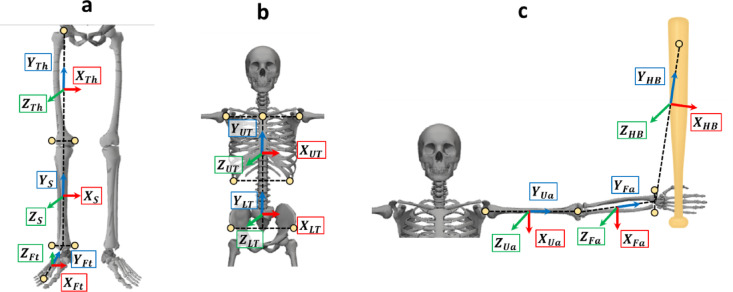
Segmental coordinate systems (a: leg, b: trunk, c: arm).

To calculate each joint angle in the experiment, a local reference frame was defined for each rigid body segment. Eight segmental coordinate systems were defined (Fig. 4) for the right-handed batters. The segmental coordinate systems are constructed using reflective markers. Figure 4 shows each segmental coordinate system for a right-handed batter. For left-handed batters, the segmental coordinate systems were defined in the same way by reversing the positive and negative signs of the $\:{Y}_{G}$ coordinates of the reflective markers on the body. Based on previous studies, the sweet spot of the bat was defined as the position which inserted 0.1 m from the attached reflective marker at the end of the bat to the bottom of the bat^19,20^. The joint angles were calculated from the hitting motions at different hitting points. The constructed model thus performs a hitting motion at different hitting points. Model construction, joint angle and segment length computations were implemented in Python (version 3.12.7; Python Software Foundation, Wilmington, DE, USA) using custom scripts.

## Calculation of the kinematic manipulability ellipsoid

The joint angle vector representing each joint angle of the rigid linkage model with 21 DOF can be expressed as $\:\varvec{\theta\:}={\left[{\theta\:}_{1},{\theta\:}_{2},\cdots\:,{\theta\:}_{21}\right]}^{{\rm\:T}}$. The position vector of the sweet spot of the bat can be expressed as $\:\varvec{r}=\left(x,y,z\right)$. The first-order differential kinematics of the manipulator can be written as

1 $$\:\dot{\varvec{r}}=\varvec{J}\left(\varvec{\theta\:}\right)\dot{\varvec{\theta\:}}\:\cdots\:$$

$\:{Z}_{Ua}\:$is the cross product of the vector from the elbow joint to wrist. $\:{Y}_{HB}$ is the vector from the wrist joint to the sweet spot of the bat. $\:{Z}_{HB}$ is the cross product of the vector from the ulnar styloid to the sweet spot of the bat and $\:{Y}_{HB}$. $\:{X}_{HB}$ is the cross product of $\:{Y}_{HB}$ and $\:{Z}_{HB}$.

where $\:\varvec{\theta\:}\in\:\:{\mathcal{R}}^{21}$ and $\:\varvec{r}\in\:{\mathcal{R}}^{3}$ denote the joint and task variables, respectively. $\:\varvec{J}\in\:\:{\mathcal{R}}^{3\times\:21}$ is the Jacobian matrix. The kinematic manipulability ellipsoid is defined as the primage of a unit sphere in the space of scaled joint velocity. Then, $\:\varvec{J}$ is then Eigen-decomposed as follows:

2 $$\:\varvec{J}=\varvec{U}\sum\:{\varvec{V}}^{{\rm\:T}}\:\cdots\:$$

where $\:\varvec{U}={\left[{\varvec{u}}_{1},{\varvec{u}}_{2},{\varvec{u}}_{3}\right]}^{{\rm\:T}}$, $\:\varvec{V}\in\:{\mathcal{R}}^{21\times\:21}$is eigenvector of $\:\varvec{J}$ and

3 $$\:\sum\:=\left[\begin{array}{ccc}{\sigma\:}_{1}&\:0&\:0\\\:0&\:{\sigma\:}_{2}&\:0\\\:0&\:0&\:{\sigma\:}_{3}\end{array}\right]\in\:{\mathbb{R}}^{3\times\:3}\:\cdots\:\:$$

From these variables, the axis of kinematic manipulability ellipsoid is $\:{\left[{\sigma\:}_{1}{\varvec{u}}_{1},\:\:{{\sigma\:}_{2}\varvec{u}}_{2},\:\:{\sigma\:}_{3}{\varvec{u}}_{3}\right]}^{{\rm\:T}}$.

The kinematic manipulability ellipsoid was calculated from the joint angles at 100 ms before the ball impact under each condition. The kinematic manipulability measure can be calculated as follows:

4 $$\:\omega\:={\sigma\:}_{1}\cdot\:{\sigma\:}_{2}\cdot\:{\sigma\:}_{3}\:\cdots\:\:$$

The kinematic manipulability measures were compared at different hitting points to assess the kinematic manipulability of the bat.

### Statistical analysis

To evaluate the magnitude of the bat’s displacement at different impact points, the kinematic manipulability indices at each hitting point were compared. The kinematic manipulability ellipsoid is represented as an ellipsoid in three-dimensional space with three mutually orthogonal principal axes. In this study, to evaluate the ease of bat control in each direction, the maximum values of the principal axes of the manipulability ellipsoid with respect to each axis of the ground coordinate system were calculated for each hitting point. Each principal axis of the manipulability ellipsoid has different components along the axes of the ground coordinate system, and the largest of these components was defined as the “maximum value along each ground coordinate system axis.” Although the kinematic manipulability reflects the volume of the kinematic-manipulability ellipsoid, it cannot assess the shape of the ellipsoid (i.e., the magnitude in each direction). Therefore, we compared the ellipsoid shape using the maximum principal axis along each direction 100 ms before impact. The components of kinematic manipulability in each direction have their own significance. The $\:{X}_{G}$ axis is the line connecting the pitcher and the catcher. Therefore, the $\:{X}_{G}$ component of the kinematic manipulability ellipsoid indicates how easily batters adjust the timing. The value of the $\:{Y}_{G}$ component of the kinematic manipulability ellipsoid indicates how easily batters displaced the bat’s sweet spot inside or outside. The $\:{Z}_{G}$ component of the kinematic manipulability ellipsoid indicates how easily the batters displaced the sweet spot in the vertical direction. In addition, the kinematic manipulability measure at 100 ms before impact were compared across different hitting points. A two-way repeated-measures analysis of variance (ANOVA) test was used to investigate the interaction and simple main effect on the maximum values of each axis component of the principal axes in the kinematic manipulability ellipsoid and kinematic manipulability measure. Height (three conditions: high, middle height, and low) and course (three conditions: inside, middle course, and outside) were used as factors. If a significant interaction was found, post-hoc multiple comparisons with Bonferroni correction were performed for height and course. The significance level was set at 5%. SPSS Statistics 28 (IBM Corp.) was used for all the statistical analyses. The normality of the distribution of data was assessed using the Shapiro–Wilk test. As a result, normality was confirmed for all datasets except two (*p* > 0.05). For the two datasets in which normality was not confirmed, visual inspection of Quantile–Quantile plots and histograms revealed no substantial skewness or kurtosis, and the analysis was therefore continued.

## Results

**Fig. 5:**
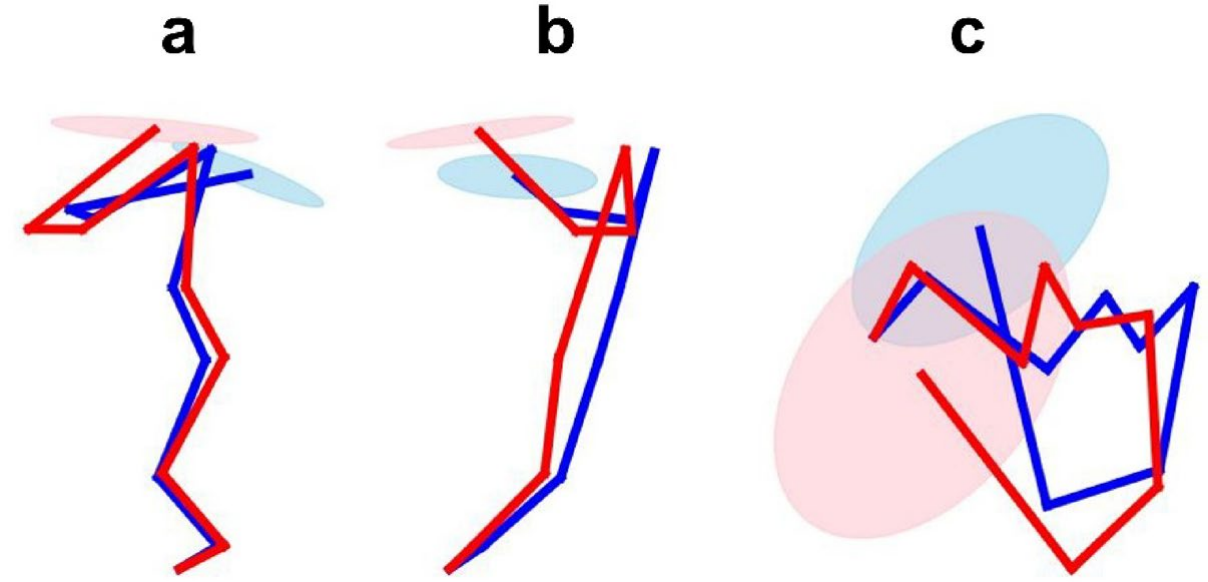
Stick pictures of a batter and kinematic manipulability ellipsoid.

Figure 5 presents the batter’s stick picture and kinematic manipulability ellipsoid at the moment when the kinematic manipulability ellipsoid was calculated. The red stick figure and ellipsoid represent the batter’s posture when hitting a low inside ball, while the blue picture represents those for a high outside.

This is the right-handed batter seen from pitcher’s view (a), side view (b), and top view (c) during the 100 ms prior to impact. The red picture represents the batter’s posture and kinematic manipulability ellipsoid when hitting a low inside, while the blue picture represents those for a high outside. Then the center of kinematic manipulability ellipsoid is the bat’s sweet spot.

**Fig. 6:**
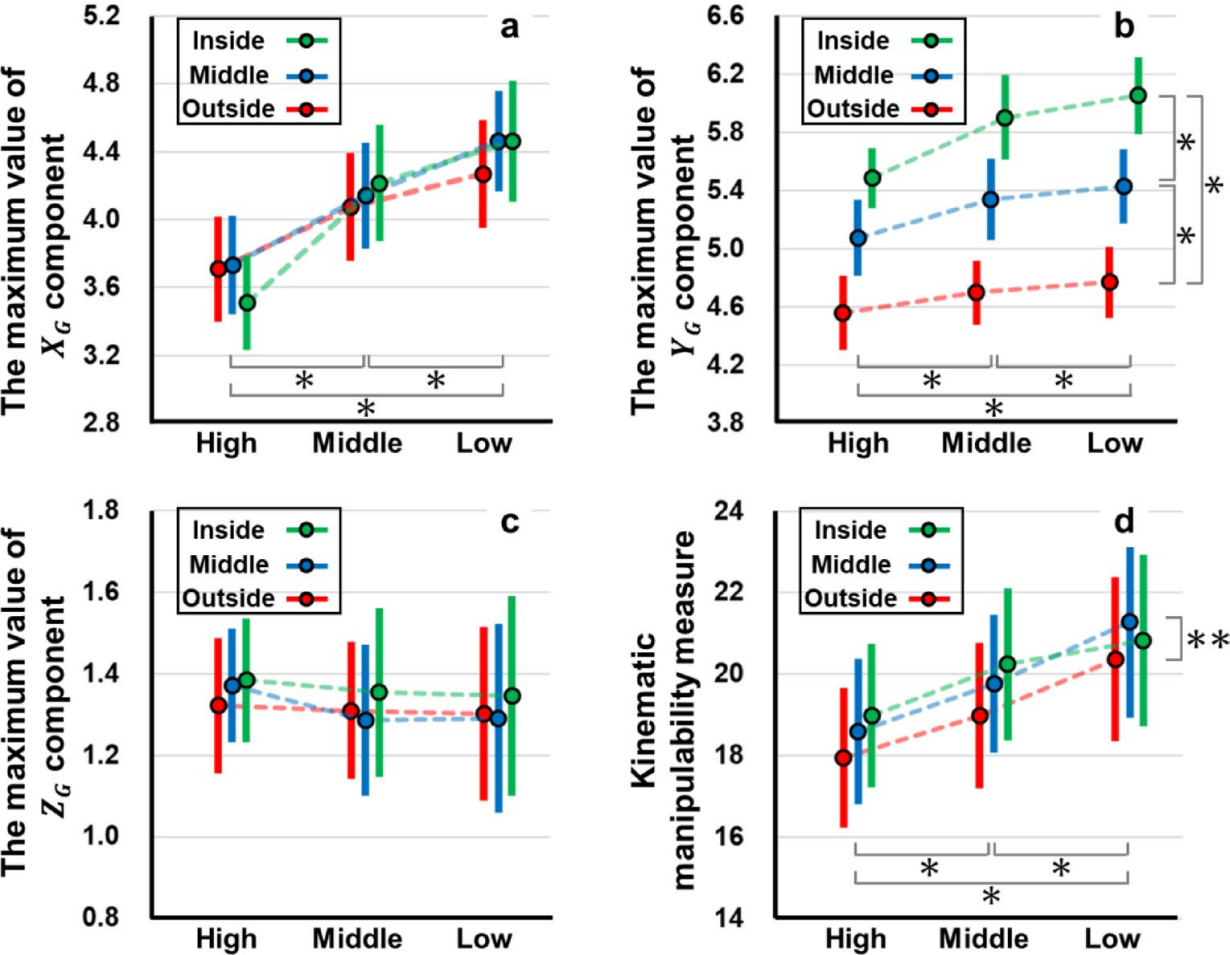
Results of the kinematic manipulability ellipsoid and the kinematic manipulability measure.

For the maximum value of the $\:{X}_{G}$ component in the principal axis of the kinematic manipulability ellipsoid at 100ms before impact, the range of the maximum and minimum of the mean values at each hitting point was 3.71–4.46. There was a significant interaction between the height and course of the impact point (F(4, 76) = 3.901, *p* = 0.021, ηₚ²= 0.170; Fig. 6a). Furthermore, there were significant self-evaluation main effects for height (F(2, 38) = 93.07, *p* < 0.001, ηₚ²= 0.830), whereas there was no significant simple main effect for the course (F(2,38) = 0.802, *p* = 0.434, ηₚ²= 0.040). Post-hoc multiple comparison tests for height showed that the $\:{X}_{G}$ component was significantly smaller at the high impact points than that at the middle height and low impact points (*p* < 0.001, t = 8.821 and t = 11.51). In addition, $\:{X}_{G}$ component at the middle height point was significantly smaller than that at the low-impact points (*p* < 0.001 t = 5.667).

For the maximum value of the $\:{Y}_{G}$ component in the principal axis of the kinematic manipulability ellipsoid at 100ms before impact (4.56–6.05), there was no significant interaction between the height and course of the impact point (F(4, 76) = 2.707, *p* = 0.208, ηₚ²= 0.125; Fig. 6b). However, significant simple main effects were observed in both groups (F(2,38) = 20.73, *p* < 0.001, ηₚ²= 0.522 and F(2,38) = 30.86, *p* < 0.001, ηₚ²= 0.914). Post-hoc multiple comparison tests for height showed that the $\:{Y}_{G}$ component was significantly smaller at the high impact points than that at the middle height and low impact points (*p* < 0.001, t = 4.911 and t = 5.386). In addition, the $\:{Y}_{G}$ component was significantly smaller at the middle height impact point than that at the lower impact point (*p* < 0.001,t = 1.872). For the course of impact point, the $\:{Y}_{G}$ component was significantly smaller at the outside impact point than that at the middle course and inside (*p* < 0.001, t = 11.82 and t = 15.56). Moreover, the $\:{Y}_{G}$ component was significantly smaller at the middle course impact points than that at the inside impact points (*p* < 0.001, t = 12.69).

For the maximum value of the $\:{Z}_{G}$ component in the principal axis of the kinematic manipulability ellipsoid at 100ms before impact (1.29–1.39), there was no significant interaction between the height of the impact point and the course (F(4, 76) = 0.438,*p* = 0.368, ηₚ²= 0.023; Fig. 6c). Furthermore, no significant simple main effects were found for the height of the impact point and the course (F(2,38) = 0.501, *p* = 0.506, ηₚ²= 0.026 and F(2,38) = 1.557 *p* = 0.061, ηₚ²= 0.076).

For the kinematic manipulability measure at 100ms before impact (18.0- 21.3), there was no significant interaction between the height of the impact point and course (F(4, 76) = 1.402,*p* = 0.368, ηₚ²= 0.069; Fig. 6d). In addition, there was no significant simple main effect for the height of the impact point (F(2,38) = 9.575, *p* = 0.080, ηₚ²= 0.335). However, a significant main effect was found for the course of the impact point (F(2,38) = 6.208, *p* = 0.009, ηₚ²= 0.246). Post-hoc multiple comparison tests for the course of the impact point that the kinematic manipulability measure at the outside impact point was significantly smaller than that the kinematic manipulability measure at the impact point in the middle course (*p* = 0.010, t = 3.188).

Each points **a** : maximum value of the $\:{X}_{G}$ component in the principal axis of the kinematic manipulability ellipsoid. **b** : the maximum value of the $\:{Y}_{G}$ component in the principal axis of the kinematic manipulability ellipsoid. **c** : the maximum value of the $\:{Z}_{G}$ component in the principal axis of the kinematic manipulability ellipsoid. **d** : kinematic manipulability measure in the different impact points. In the figures, * and ** show the significant differences between indicating condition in the post-hoc Bonferroni multiple comparison (* : *p* < 0.001, ** : *p* = 0.010).

## Discussion

The objective of this study was to compare the kinematic manipulability of baseball hitting at different impact points. For the maximum value of the $\:{X}_{G}$ component in the principal axis of the kinematic manipulability ellipsoid, there was a significant interaction between the height of the impact point and course (*p* = 0.021; Fig. 6a). Moreover, the maximum value of the $\:{X}_{G}$ component decreased significantly higher hitting point (*p* < 0.001). The $\:{X}_{G}$ axis is the line connecting the pitcher and the catcher. Therefore, batters can easily displace the sweet spot of the bat toward the pitcher or catcher side with a high value of kinematic manipulability. To hit the ball, the batter needs to adjust the timing and control the impact point in $\:{X}_{G}$^21,22^. These results indicate that timing adjustments are facilitated when the impact point is low within the strike zone. Consequently, batters may decrease the probability of a swing strike while sustaining effective contact by selectively targeting low pitches. From the pitcher’s perspective, throwing high pitches can reduce the batter’s possibility of establishing contact by complicating the timing adjustments. Previous studies^21,22^ showed that batters adjusted the timing by modulating the timing of the stride-foot stepping movement and the forward weight shift. The peak ground reaction force under the stride foot occurred 105–113 ms before contact. In this study, the kinematic-manipulability ellipsoid was evaluated 100 ms before impact. Thus, this kinematic-manipulability analysis may serve as a kinematic measure of the ease with which hitters adjust their timing after stepping.

For the maximum value of the $\:{Y}_{G}$ component in the principal axis of the kinematic manipulability ellipsoid, the $\:{Y}_{G}$ component significantly decreased with higher impact points (*p* < 0.001) and further decreased toward the outside (*p* < 0.001). The $\:{Y}_{G}$ component is in the direction from left to right of the batter’s box (inside and outside). The maximum value of the $\:{Y}_{G}$ component in the principal axis of the kinematic manipulability ellipsoid indicates how easily batters displaced the bat’s sweet spot inside or outside. In the lower strike zone compared with the higher strike zone, it is possible that batters may correct the horizontal gap between the bat and ball. In the same way, batters may correct the horizontal gap between the bat and ball when hitting the ball at inside. In the game, batters may decrease the probability of a swing strike while sustaining effective contact by selectively targeting lower or more inside pitches. Moreover, batters can improve their contact capability by setting up closer to the home plate in the batter’s box, thus shifting the perceived pitch locations toward the inside of the box.

For the maximum value of the $\:{Z}_{G}$ component on the principal axis of the kinematic manipulability ellipsoid, no significant interactions were observed between the height and course of the hitting area. The $\:{Z}_{G}$ component is in the vertical direction. The maximum value of the $\:{Z}_{G}$ component on the principal axis of the kinematic manipulability ellipsoid indicates the ease with which the batters displace the sweet spot in the vertical direction. This indicates that the batters’ ability to displace the bat vertically remains relatively stable regardless of the pitch location. However, the $\:{Y}_{G}$ values exceeded the $\:{Z}_{G}$ values on the principal axis of the kinematic manipulability ellipsoid (Fig. 6b, c). This indicates that the batters can more easily displace the bat’s sweet spot horizontally than vertically. Figure 5 illustrates the body posture of the participant and the kinematic-manipulability ellipsoid as observed by the pitcher 100 ms before impact. The infinitesimal angular displacements of the joints displace the end point in a direction perpendicular to the longitudinal axis of the segment (tangential direction). In Fig. 5, the lower limb and trunk segments are oriented perpendicular to the ground. Consequently, the bat’s sweet spot is more likely to be displaced in the horizontal direction. In this game, batters are expected to improve their contact rates when they target pitches with small vertical breaks (i.e., those whose trajectories shift less vertically). In training, focusing on handling vertically breaking pitches should help improve the overall hitting skill. Conversely, inducing swing strikes with vertically breaking pitches may be easier, and acquiring such pitches is expected to enhance the pitcher’s pitch performance. In a previous study, Higuchi et al. investigated the spatial relationship between the sweet spot of a bat and a ball at the point of impact^23^. They reported that the standard deviation of the impact point on the long axis of the bat exceeded that on the perpendicular axis. In the posture at impact, $\:{Y}_{G}$ and $\:{Z}_{G}$ are almost on the same axis as the long axis of the bat and on the perpendicular axis. Thus, the direction with greater variability in the impact points on the bat corresponds to the direction with higher bat kinematic manipulability. The potential to widely displace the sweet spot of the bat may cause greater variability in the impact points on the bat. This suggests that the magnitude of kinematic manipulability is not necessarily related to repeatability or accuracy.

Regarding the kinematic manipulability measure, the kinematic manipulability measure for hitting the outside area was significantly smaller than that for hitting the middle course (*p* = 0.010). The kinematic manipulability measure is proportional to the volume of the kinematic manipulability ellipsoid and indicates how easy it is to arbitrarily change the position and orientation of the end effector at the tip of the manipulator. Considering the results of the statistical analysis, it is suggested that batters may be able to easily manipulate the bat when swinging at a pitch over the middle course of the plate compared to a pitch on the outside of the strike zone. Therefore, there is a better chance that the batter can make contact with the ball when the pitch is over the middle course of the plate compared to when the pitch is over the outside of the strike zone. Differences in the bat speed 100 ms before impact across the impact locations were examined. The velocities of the bat’s end point 100 ms before impact were 9.26 ± 0.99 and 9.39 ± 1.19 m/s for the inside-low and outside-high conditions, respectively. In both conditions, the values correspond to approximately 27% of the bat speed at impact. Thus, the bat’s kinematic manipulability was affected by postural differences arising from the impact locations 100 ms before impact when the bat was being similarly accelerated. In the hitting direction, although the present study did not fully record the batted-ball direction, if the hitters imagined a specific hit direction, then the resulting posture changes might alter the manipulability indices.

**Fig. 7:**
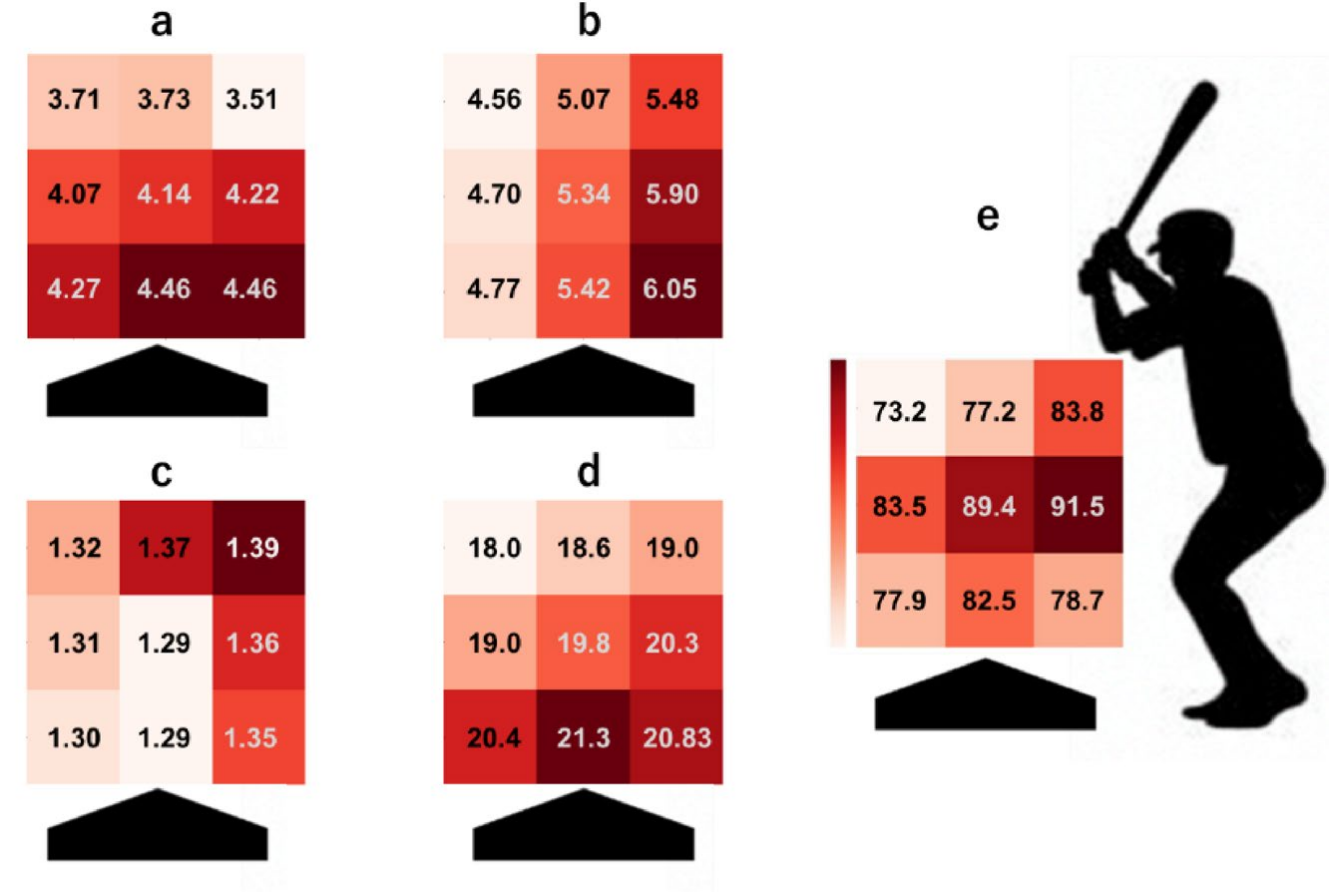
The comparison of kinematic manipulability to the ball contact percentages for each area.

To compare the findings of this study to the actual results of baseball games, Fig. 7e illustrates the percentage of swings that contact the ball (contact ball/swing) [%] for each strike area in the 2021–2023 Major League Baseball (MLB) seasons. This was calculated from Baseball Savant, which is available as open-source data from MLB. Previous studies have used this system^24,25^. A comparison of the kinematic manipulability of the bat and the percentage of swings that contact the ball showed similar trends. In actual baseball games, there is a trend that the percentage of swing contact with the ball is lower in the high strike area compared to the middle height and low strike area at the same course. This is consistent with the analysis of the kinematic manipulability ellipsoid (Fig. 7a, b, d, e). Moreover, the percentage at which the swing contacts the ball was higher in the middle course of the strike area than on the outside at the same height. This is consistent with the analysis of the kinematic manipulability of the bat (Fig. 7b, d). The percentage at which the swing contacts the ball was higher in the middle height areas of the strike zone than in the upper and lower areas of the strike zone with the same course. This result differs from the kinematic manipulability analysis in this study (Fig. 7a, b, c, d). This difference may have been caused by hitting stationary ball in the experiment. In actual batting, batters must swing the bat while predicting the point of impact based on the speed and trajectory of the pitch. As a result, the batter’s swing motion shows variability. For this reason, the posture 100 ms before impact, which is used to calculate the kinematic manipulability ellipsoid, may differ between the actual baseball swing and this experiment. In addition, the results of this study may have been influenced by the choice of 100 ms before impact as the analysis time point. The posture at this specific time point when hitting a given course might resemble the posture at a different time point when hitting another course. These factors should be regarded as important limitations of the present study. This study employed the hitting of stationary balls to clearly define the impact points. This approach allowed for an accurate evaluation of the batter’s kinematic manipulability at different impact locations. The ability to assess manipulability across various impact points may provide meaningful insights for batters in formulating their hitting strategies. In the future, it will be valuable to obtain data from experimental environments that closely approximate actual baseball hitting tasks, such as hitting pitches with different trajectories and movements (sliders, curves, and split-fingered fastballs). Evaluating the temporal changes in posture and manipulability during the swing under such conditions will provide more comprehensive insights.

One limitation of this study is that the kinematic manipulability ellipsoid does not take dynamics into account. During the swing, torque is exerted on the joints of the batter to control and accelerate the bat until it makes contact with the ball. The kinematic manipulability ellipsoid is a quantitative measure with which the position of the end effector can be changed arbitrarily with an infinitesimal angular displacement of the system. The key to defining such ellipsoids is the Jacobian manipulator, which describes the mapping from joint space to task space. These kinematic manipulability indices were calculated from a static perspective only. In this study, the magnitude of kinematic manipulability was determined by the batter’s joint angles and segment length, except for torque, velocity and acceleration. Previous studies have proposed an approach that takes the joint torque into account^26,28^. In future research, the manipulability of the bat will be described in more detail using a kinetic manipulability index that takes dynamics into account. Nevertheless, this study gives useful insights by applying static kinematic manipulability to baseball hitting in a new way. It helps batters understand batting strategies and improve performance from a biomechanical and robotic point of view.

The points represent the mean values of the manipulability index for each condition, and the error bars indicate the 95% CI. **a** : maximum value of the $\:{X}_{G}$ component in the principal axis of the kinematic manipulability ellipsoid. **b** :the maximum value of the $\:{Y}_{G}$ component in the principal axis of the kinematic manipulability ellipsoid. **c** :the maximum value of the $\:{Z}_{G}$ component in the principal axis of the kinematic manipulability ellipsoid. **d** : kinematic manipulability measure in the different impact points. **e** : ball contact percentage for each area during the 2021–2023 Major League Baseball seasons [%]. Darkly colored areas indicate areas with high ball contact.

Another limitation is the exclusion of the trailing arm in the kinematic manipulability analysis. This model assumes that the lead arm determines the bat’s reachable positions, simplifying the batter’s two-arm system into a single serial-link.

system. In reality, both arms contribute to bat control. This simplification was adopted considering that kinematic manipulability is defined for open-chain systems. The simplification of the model made it possible to observe how different body postures influence the directional characteristics of bat control. As a result, it helped to improve the understanding of the shape of the kinematic manipulability ellipsoid. Future research could incorporate a dual-arm system or closed-chain manipulability analysis to enable a more comprehensive assessment of bat control.

This study presents several limitations that should be acknowledged. First, the results may have been affected by the participants’ competitive level. Previous studies reported that the kinematic characteristics during swinging differ depending on the competitive category (e.g., junior high school, high school, and college) and hitting performance level^29,30^. These findings suggest that differences in kinematic manipulability exist at different competition levels. In fact, some differences were observed between the present findings and data reported for MLB players. Hence, future studies should clearly categorize participants by competitive level and compare the manipulability indices across these groups. Furthermore, whether high kinematic manipulability results in better hitting outcomes (such as contact rate, ball speed, and flight distance) remain ambiguous. Future studies should statistically examine this relationship through comparisons across trials and between subjects. As an example, future studies should examine whether swings with high kinematic manipulability can consistently yield successful hitting outcomes. This would clarify the validity and practical utility of the manipulability index.

The findings of this study may have practical implications for the development of targeted batting methods. Moreover, batters can benefit from examining movements associated with higher kinematic manipulability at specific hitting points, thus potentially improving their batting performance during games. For example, identifying hitting points with higher kinematic manipulability may help coaches design training drills that emphasize optimal bat control in specific strike-zone regions. Future studies should investigate whether targeted training based on kinematic-manipulability analysis effectively improves batting performance in actual game situations.

## Conclusions

This study compared the kinematic manipulability of baseball hitting at different impact points. These results provide valuable insights for strategy formulation, as they suggest the balls that the batter should hit. Our findings suggest that timing adjustment is easier when the pitch is low in the strike zone. In addition, batters may correct the horizontal gap between the bat and ball more easily in the lower or inside strike zone. The results were generally consistent with the actual baseball results. In the future, it will be necessary to obtain data from experimental environments that are closer to actual baseball hitting tasks.

## Supplementary Information

Below is the link to the electronic supplementary material.

Supplementary Material 1

## Data Availability

The datasets generated during and/or analyzed during the current study are available from the corresponding author on reasonable request.
